# Motor Development of Children and Adolescents Aged 8–16 Years in View of Their Somatic Build and Objective Quality of Life of Their Families

**DOI:** 10.2478/v10078-011-0021-1

**Published:** 2011-07-04

**Authors:** Daniel Puciato, Władysław Mynarski, Michał Rozpara, Zbigniew Borysiuk, Renata Szyguła

**Affiliations:** 1 Faculty of Physical Education and Physiotherapy, Opole University of Technology, Poland.

**Keywords:** motor development, somatic traits, quality of life, children, adolescents, parents

## Abstract

The differences in human motor development are determined by predispositions and living conditions. The aim of the present study was to examine relationships between motor fitness of children and adolescents aged 8–16 years (277 boys and 247 girls), and their somatic build and quality of life of their families. Body height, body mass and skinfold thickness were measured. On the basis of these measurements body mass index (BMI), Rohrer’s index and lean body mass (LBM) were calculated. The subjects’ physical fitness was also assessed with motor tests: speed of arm movement (plate tapping), agility (10 x 5 m shuttle run), explosive strength of the legs (standing broad jump), trunk strength (situps), explosive strength of the trunk and shoulder girdle (1-kg medicine ball throw), and flexibility (sit and reach) regarded as a morpho-functional predisposition of motor abilities. The standing broad jump results were then used to calculate maximal anaerobic power (MPA). The examination was completed with a questionnaire survey of the children’s parents concerning their families’ quality of life. On the basis of the parents’ answers to the questionnaire, two quality of life indices were constructed: objective quality of life index and subjective quality of life index. Due to the wide age bracket of subjects the sample was divided into two age groups: 8–12 and 13–16-year-olds. The relationships between subjects’ motor development, somatic traits and their families’ quality of life were examined with the use of multivariate comparative analysis. The level of motor development of studied children was more strongly determined by their somatic build than the quality of life of their families. The most important somatic determinants of the subjects’ motor abilities were body height and subcutaneous adiposity. These determinants primarily affected speed and strength abilities of younger school children. Objective quality of life of children’s families determined the development of some strength abilities in children aged 8–12 years. No correlations between the subjects’ motor development and subjective quality of life of their families were found.

## Introduction

Differences in ontogenetic biological development have two main sets of causes. The first are individual, genetically programmed predispositions to the pace (dynamics) and level (kinetics) of biological development as well as living conditions in childhood and adolescence. The others are include multidimensional interactions and interdependencies between the genotype and the environment. In consequence, the genotype determines the patterns of body’s reactions to specific environmental conditions, and the environment determines what parts of these norms can be used by particular genotypes (Giagazoglou et al., 2007, Gültekin et al., 2006, Venetsanou and Kambas, 2010). The biological development of the human body is also stimulated by well-organized and age suitable physical activity, which positively influences the development of the skeletal, muscular, cardiovascular, respiratory and many other systems (Ekelund et al., 2001, Kemper et al., 2001).

The motor development of children and adolescents, manifested by their physical fitness, is a complex of genetic, somatic, motor and behavioral components. These components are also affected by such social and family factors as income and education level, parents’ occupaton, place of residence, family size or ethnicity. These factors can also indirectly – in combination with socio-economic conditions and lifestyles – modify the motor development of youth (Bouchard et al., 1994, Suliga, 2009).

The most popular criteria of social and family status are currently objective and subjective quality of life indices. Objective quality of life is the total of possessed material goods, services, states and situations constituting the general well-being of individuals. Subjective quality of life is related to the level of one’s satisfaction or dissatisfaction with their material goods and services and with different areas of human life (Borys and Rogala, 2008). The necessity to discriminate between both types of quality of life assessment results from the complex relations between them. According to Campbell, there is no unidirectional relationship between objective quality of life and life satisfaction level. In other words, by no means is an improvement in one’s social and family status related to one’s increased satisfaction with one’s life (Dittmann and Goebel, 2010, Veenhoven, 2005, Welzel and Inglehart, 2010).

A number of research studies have been concerned with the somatic and environmental determinants of motor development of children and adolescents (Ignasiak and Sławińska, 1999, Mynarski et al., 2007, Saavedra et al., 2008). Few of them, however, considered the socio-economic status of children’s families in terms of objective and subjective quality of life. In regard to this shortage the present study aims to examine somatic and socio-economic determinants of motor fitness of children and adolescents aged 8–16 years. The following research hypotheses were formulated:

1. Somatic traits in boys and girls have a statistically significant effect on their level of motor development.
2. The level of development of motor abilities of children may be related, to a different degree, to the objective and subjective quality of life of their families.

## Material and methods

The study material consisted of data obtained from primary and middle school students from Jedlina-Zdrój in Poland in 2004. In total, 524 school students (277 boys and 247 girls) aged 8 to 16 years took part in the study. The study was approved by the respective local bioethics committee.

The subjects’ somatic and motor development was measured with the use of direct observation, anthropometric measurements and motor fitness tests. Body height was measured with an anthropometer, body mass with a scale and skinfold thickness with calipers in three locations: abdomen (between the umbilicus and the anterior superior iliac spine), subscapula (beneath the edge of the shoulder blade) and triceps (posterior midline of the upper arm). Using the obtained data the subjects’ body mass index (BMI), Rohrer’s index and lean body mass (LBM) were calculated. The following fitness tests were carried out: speed of arm movement (plate tapping), agility (10 x 5 m shuttle run), explosive strength of the legs (standing broad jump), trunk strength (sit-ups), explosive strength of the trunk and shoulder girdle (1-kg medicine ball throw), and flexibility (sit and reach) regarded as a morpho-functional predisposition of motor abilities. The standing broad jump results were used to calculate maximal anaerobic power (MPA).

The subjects’ examination was completed with a questionnaire survey of their families’ quality of life made by the parents. A modified version of the quality of life questionnaire (Rusnak and Kozyra, 2001) was used. A pilot study was conducted on a small group of subjects before the main questionnaire survey. The survey reliability index was 0.90. On the basis of survey data the objective and subjective quality of life indices were constructed. The objective quality of life index was based on the subjects’ answers to the questionnaire items regarding the number of children in the family, parents’ education, parents’ occupation, family type (complete, incomplete) and ways of spending vacation, with the use of multivariate comparative analysis (MCA) by way of measure of development. The value of the subjective quality of life index was an arithmetic mean calculated from answers to ten questionnaire items on a visual analogue scale (1–7). The respondents were to make a general evaluation of their quality of life: the extent to which their life was interesting, easy, diversified, valuable, fulfilled and accomplished, and to which they themselves were happy, needed and esteemed (Rusnak and Kozyra 2001).

Due to the wide age bracket of the sample and differences in somatic and motor development, the subjects were divided into two age groups: 8–12-year olds and 13–16-year-olds. To examine the somatic and socio-economic determinants of subjects’ motor development, a multivariate stepwise regression analysis was applied. The level of statistical significance was set at α = 0.05.

## Results

The analysis of results showed that arm movement speed in children aged 8–12 was most strongly determined by their body height. The model of regression for arm movement speed also included such exogenous variables as subcutaneous adiposity, subjective quality of life (in boys) and objective quality of life (in girls). However, the relationships between arm movement speed and these variables were statistically non-significant. Since the coefficients of determination (R^2^) for arm speed movement amounted to 0.42 in boys and 0.34 in girls, the specific models only partially explained the variability of arm movement speed in children aged 8–12 years. Other exogenous variables should be sought to explain this motor characteristic fully. The analysis revealed that better plate tapping results were obtained by taller boys and girls (Tabs. 2, 3).

In the older group (13–16 years), there was a statistically significant and unidirectional relationship between body mass in boys and body height in girls. This means that the best plate tapping test results were attained by the heaviest boys and the tallest girls. In the regression analysis, the relationships between arm movement speed and socio-economic variables: objective quality of life index (in boys) and subjective quality of life index (in girls) were statistically non-significant (Tab. 4, 5).

It should be noted that the coefficients of determination (R^2^) in the given models of regression were higher in the younger group of subjects (8–12 years) than in the older one (13–16 years). This means that the exogenous variables determined arm movement speed to a greater extent in children than in adolescents (Tab. 2–5).

In the groups of children (girls only) and adolescents agility was significantly determined by body height and skinfold thickness. Also, the objective quality of life index,results with the exception of girls aged 13–16, corrected, on the verge of statistical significance, with the subjects’ shuttle run test results (Tab. 2–5).

In the group of children aged 8–12 years, strength test results were related with body height and subcutaneous adiposity. The boys under study also displayed a unidirectional relationship between the body mass index (BMI) and explosive strength of the trunk and shoulder girdle. As far as variables determining the socio-economic status of subjects’ families are concerned, a higher level of explosive strength of the legs in boys was significantly determined by a higher objective quality of life index. In the group of girls, the higher objective quality of life index was significantly related to lower explosive strength of the trunk and shoulder girdle (Tab. 2, 3).

The determinants of strength abilities in adolescents under study (13–16 years) were more liable to change. The explosive strength of the legs, maximal anaerobic power and trunk strength in the older boys were related to their skinfold thickness. In the same group of boys the explosive strength of the trunk and shoulder girdle were determined by body mass. In girls, explosive strength of the trunk and shoulder girdle were significantly affected body height (Tab. 4, 5). It should be noted that in the group of 13–16-year-old girls none of the exogenous variables determined trunk strength.

In the studied sample, maximal anaerobic power (MAP) was most significantly determined by the analyzed somatic traits and indices as well as quality of life indices. The regression equations describing the MAP variability featured the highest coefficients of determination (R^2^). In younger boys, all three exogenous variables determining MAP, i.e. body height, LBM and objective quality of life index, were statistically significant. MAP was correlated with subjects’ LBM, body height and objective quality of life index. In younger girls, the objective quality of life index was “replaced” as a determinant of MAP variability by body mass (Tab. 2, 3). Among adolescent boys MAP was significantly determined by body mass and total thickness of three skinfolds. Higher maximal anaerobic power was characteristic of the boys under study, who had greater body mass and less subcutaneous fat. In adolescent girls studied was the best determinant of MAP body mass (Tab. 4, 5).

In the studied sample, with the exception of girls aged 13–16 years, the high level of flexibility (motion range of spinal and hip joints) was related with low adiposity and high lean body mass. In the group of adolescent boys, also a low level of flexibility was noted in shorter subjects (Tab. 2–4). None of the studied exogenous variables was related to flexibility in girls (Tab. 5).

## Discussion

In the sample of children and adolescents studied the level of motor development was significantly determined by certain somatic traits. A more advanced development of speed and strength variables was observed in tall children with less subcutaneous fat tissue. This was also noted by other authors, who indicated that better motor test scores often derived from more advanced processes of growth and maturation accompanied by qualitative development of body structures and functions (Ignasiak and Sławińska, 1999, Malina et al., 2004, Suchomel, 2005). This is also confirmed in the present study, where children aged 8–12 displayed much more statistically significant relationships between physical fitness components and morphological build, as compared with their older counterparts (13–16 years) in whom the pubertal growth spurt may interfere with the structure of their motor potential. Thicker subcutaneous fat, especially visible in pubescent girls, and thus a lower level of lean body mass determine lower motor test results (Mynarski et al., 2007, Suchomel, 2005), which remains in accordance with the results of these study.

In the present study objective quality of life affected indirectly some strength abilities in the group of younger children. In boys, the higher value of the objective quality of life index is related to greater explosive strength of the legs and maximal anaerobic power. In girls, a higher socio-economic status is related to lower results of the medicine ball throw test. Socio-economic factors did not then exert a significant impact on the motor development of adolescents aged 13–16 years. Other authors showed, however, that children’s motor development can be modified by socio-economic factors – so-called civilization and cultural modifiers – regardless of their somatic build and physical activity level (Giagazoglou et al., 2007, Ignasiak et al., 2002, Kimhi, 2003, Mészáros et al., 2008, Pavón et al., 2010). Some elements of the socio-economic status may nevertheless affect the development of the human body. For example, thanks to a high level of income in a family, children can be assured proper accommodation, nutrition, good sports equipment and organized sports and recreational activities (Brett Schneider and Naul 2004). Parents’ education can affect their children’s hierarchy of values, hygiene and increased parental awareness of proper upbringing, nutrition, and active leisure (Piko and Keresztes, 2008, Puciato, 2010a, 2010b). In the present study objective quality of life only affected the motor development of younger children, which can be related to the fact that the impact of socioeconomic factors on ontogentic development is different in different stages. Research studies show that the impact is greater if the developmental processes are faster (Lindgren et al., 1994, Puciato, 2010b). This is evidence of the important role of the environment in the stimulation of young school children’s development. The study results show that the development of motor abilities in older children is more related to their physical activity than with their somatic build or living conditions (Mynarski et al., 2007). The most important effect of physical activity on health is an optimal development of motor fitness. It is commonly regarded as a positive measure of health (Bouchard et al., 1994, Mynarski et al., 2007). The biological effectiveness of physical activity depends on its form, frequency, volume and intensity. The higher these variables are, the more the undertaken physical efforts affect the development of physical fitness, increase oxygen intake and contribute to the adaptive processes of the body (Crews et al. 2004, Ekelund, 2001, Mynarski et al., 2007, Mynarski et al., 2009). In the context of proper functional development and health condition of the young generation, the study results pointing to a low level of physical activity of young people are quite disturbing. According to Drygas et al. (2007), almost one half of Polish adolescents do not undertake any physical exercise in their free time.

The results of the present study also point to a higher level of impact of socio-economic factors on the motor development of boys, similar to results by other authors (Ignasiak et al., 2002). It should be remembered that such factors as parents’ occupation and education, income and quality of life affects the children’s motor development only indirectly. This can be confirmed by results of some studies showing that positive changes in the somatic development of children from families of high social status do not go hand in hand with their motor development (Mynarski et al., 2007). Also in the present study lower results of explosive strength of the arms were attained by younger girls from families with a high objective quality of life index. Mleczko (1991) in his study of urban children and youth from Cracow observed that social stratification is not always a discriminating factor of their functional development. He concluded that the higher level of functional development of children from lower social classes, mainly girls, had been most likely the result of the impact of cultural patterns. The motor potential of boys and girls with a better level of somatic development and social status is constrained by these patterns, mainly due to limited physical activity and passive lifestyle. Spontaneous physical activity is more and more often characteristic of children and adolescents from lower social classes (Piko and Keresztes, 2008). For this reason, American researchers acknowledge environmental determinants of physical activity, but not of motor fitness which results from this activity (Brownson et al., 2000, King et al., 2000, Seefeldt et al., 2002). It can be thus concluded that good family conditions in children’s life not always positively influence their functional development, but usually positively affect their somatic development. This observation is confirmed by the results of the present study of somatic and socio-economic determinants of motor development of children and adolescents.

In the present study subjective quality of life of families was not a statistically significant determinant of the children’s motor development. Therefore, it seems that objective living conditions rather than their subjective evaluation are more significant modifiers of the developmental processes in young people (Puciato, 2010a, 2010b, 2010c).

Considering the number and diversity of determinants of the development of motor abilities, the assessment of their strength and direction is a complex and difficult process. Studies on the motor development of young people, in particular early school age children, should not therefore ignore their morphological build and socio-economic factors related to their families’ status. It should be remembered that these factors can both stimulate and inhibit (biologically and socially) the development of motor fitness (Mleczko, 1991, Mynarski et. al., 2007). Studies on determinants of motor development of older school children should involve lifestyle components, first of all, their and their parents’ level of physical activity. In Poland under new economic and social conditions this factor will play a very important role in the development of motor fitness of the young generation. It should be kept in mind that the level of physical activity in childhood and early adolescence determines the intensity and quality of physical activity in adult life.

## Conclusions

1. The level of motor development of Polish children city inhabitants, is more significantly determined by their somatic build than the quality of life of their families
2. The most important somatic determinants of children and youth motor abilities are body height and subcutaneous adiposity. These determinants primarily affect speed and strength abilities of younger school children.
3. Objective quality of life of children’s families determines the level of development of some strength abilities in younger children. No relationships between the subjects’ motor development and subjective quality of life of their families were found.

## Figures and Tables

**Table 1:** Number of subjects with regard to sex and age

**Sex**	**Age [years]**								
	**8**	**9**	**10**	**11**	**12**	**13**	**14**	**15**	**16**
boys	29	32	41	20	32	31	37	29	26
girls	35	36	28	22	29	25	23	27	22
total	64	68	69	42	61	56	60	56	48

**Table 2:** Regression analysis of correlations between motor abilities, somatic traits and objective and subjective quality of life indices in boys aged 8–12.

Exogenous variable	Endogenous variable						
	Arm movement speed (plate tapping)	Agility (10x5m shuttle run)	Explosive strength of the legs (standing broad jump)	Trunk strength (sit-ups)	Explosive strength of trunk and shoulder girdle (1kg medicine ball throw)	MAP	Flexibility (sit and reach)
Body height	**−0.677**	**−0.635**	**0.638**	**0.581**	**0.702**	**0.309**	
Body mass		0.302					
BMI					**0.488**		0.233
Rohrer’s index								
∑ of skinfold thickness	0.123		**−0.437**	**−0.220**	**−0.588**		**−0.455**
LBM						**0.605**	
Objective quality of life index		−0.177	**0.216**			**0.114**	
Subjective quality of life index	0.110			−0.090			0.136

R	0.649	0.494	0.685	0.543	0.727	0.924	0.308
R^2^	0.422	0.244	0.469	0.295	0.529	0.854	0.095
F	22.620	9.992	27.370	13.002	34.862	181.37	3.255
p	**0.000**	**0.000**	**0.000**	**0.000**	**0.000**	**0.000**	**0.025**

**Table 3:** Regression analysis of correlations between motor abilities, somatic traits and objective and subjective quality of life indices in girls aged 8–12.

Exogenous variable	Endogenous variable						
	Arm movement speed (plate tapping)	Agility (10x5m shuttle run)	Explosive strength of the legs (standing broad jump)	Trunk strength (sit-ups)	Explosive strength of trunk and shoulder girdle (1kg medicine ball throw)	MAP	Flexibility (sit and reach)
Body height	**−0.538**	**−0.460**	**0.704**	**0.538**	**0.813**	**0.391**	
Body mass						**0.313**	
BMI							
Rohrer’s index							
∑ of skinfold thickness		**0.193**	**−0.257**	−0.087	**−0.131**		
LBM						0.252	**0.195**
Objective quality of life index	−0.141	−0.107			**−0.179**		
Subjective quality of life index							0.150

R	0.587	0.497	0.697	0.527	0.773	0.924	0.252
R^2^	0.345	0.247	0.486	0.277	0.597	0.853	0.064
F	26.849	11.047	48.187	19.588	49.947	196.17	3.468
p	**0.000**	**0.000**	**0.000**	**0.000**	**0.000**	**0.000**	**0.034**

**Table 4:** Regression analysis of correlations between motor abilities, somatic traits and objective and subjective quality of life indices in boys aged 13–16.

Exogenous variable	Endogenous variable						
	Arm movement speed (plate tapping)	Agility (10x5m shuttle run)	Explosive strength of the legs (standing broad jump)	Trunk strength (sit-ups)	Explosive strength of trunk and shoulder girdle (1kg medicine ball throw)	MAP	Flexibility (sit and reach)
Body height		**−0.304**	0.366				**−0.444**
Body mass	**−0.375**		0.308		**0.750**	**1.144**	
BMI						−0.187	
Rohrer’s index							
∑ of skinfold thickness		**0.289**	**−0.342**	**−0.351**	−0.201	**−0.164**	
LBM							**0.792**
Objective quality of life index	−0.165	−0.214		0.179			0.146
Subjective quality of life index				0.156	−0.128		

R	0.450	0.438	0.606	0.399	0.671	0.921	0.537
R^2^	0.202	0.191	0.367	0.159	0.451	0.848	0.288
F	8.634	5.290	12.940	4.237	18.328	124.910	9.032
p	**0.000**	**0.002**	**0.000**	**0.008**	**0.000**	**0.000**	**0.000**

**Table 5:** Regression analysis of correlations between motor abilities, somatic traits and objective and subjective quality of life indices in girls aged 13–16.

Exogenous variable	Endogenous variable						
	Arm movement speed (plate tapping)	Agility (10x5m shuttle run)	Explosive strength of the legs (standing broad jump)	Trunk strength (sit-ups)	Explosive strength of trunk and shoulder girdle (1kg medicine ball throw)	MAP	Flexibility (sit and reach)
Body height	**−0.355**	**−0.484**	−1.706		**0.432**	−0.663	
Body mass					0.277	**1.998**	
BMI			5.979				
Rohrer’s index			−6.044			−1.038	
∑ of skinfold thickness		**0.385**					
LBM							
Objective quality of life index							
Subjective quality of life index	0.197						

R	0.339	0.530	0.487		0.657	0.842	
R^2^	0.115	0.281	0.237		0.431	0.709	
F	3.697	11.136	5.817		21.629	45.59 3	
p	**0.031**	**0.000**	**0.001**		**0.000**	**0.000**	
